# Segmental arterial mediolysis accompanied by renal infarction and pancreatic enlargement: a case report

**DOI:** 10.1186/1752-1947-6-307

**Published:** 2012-09-18

**Authors:** Nobuhisa Ito, Go Kuwahara, Yuta Sukehiro, Hiromitsu Teratani

**Affiliations:** 1 Department of Cardiovascular Surgery, Fukuoka University School of Medicine, 7-45-1 Nanakuma, Jonanku, Fukuoka, 814-0180, Japan

**Keywords:** Segmental arterial mediolysis, Renal infarction, Acute abdomen

## Abstract

**Introduction:**

Due to recent advances in imaging diagnostic techniques, there are an increasing number of case reports of segmental arterial mediolysis. However, there are only a limited number of reports on segmental arterial mediolysis-related abnormalities of abdominal organs other than the intestine. This report describes a case of segmental arterial mediolysis accompanied by abnormalities of abdominal organs without clinical symptoms.

**Case presentation:**

A 52-year-old Japanese man with hematuria and no prior medical history was referred to a urologist and was diagnosed as having urinary bladder cancer. He underwent trans-urethral resection of the bladder tumor and intra-vesical instillation therapy, which was followed by observation. During follow-up, although no abdominal symptoms were observed, an abdominal computed tomography scan revealed a dissection of the superior mesenteric artery. A false lumen partially occluded by a thrombus was located distal to this occlusion. The lumen was irregularly shaped with narrow and wide sections. Similar irregularities were also observed in the wall of the inferior mesenteric artery. Arterial dissection with thromboembolism in the left renal artery and renal infarction was also observed. Follow-up computed tomography after two months revealed an enlargement of the pancreatic tail adjacent to the splenic artery. Follow-up three-dimensional computed tomography showed gradual re-expansion of the true lumen of the superior mesenteric artery, improvement in arterial wall irregularities, and a reduction in the pancreas enlargement and renal infarction. Over the following 15 months, these changes gradually normalized. On the basis of the vascular changes in multiple arterial systems that resolved spontaneously, we considered that the lesions were associated with segmental arterial mediolysis.

**Conclusions:**

We present a rare case of segmental arterial mediolysis accompanied by abnormalities of abdominal organs without clinical symptoms. Three-dimensional computed tomography was useful for follow-up evaluation in our patient.

## Introduction

Segmental arterial mediolysis (SAM) is a condition characterized by segmental lysis of the tunica media of muscular arteries and subsequent formation of aneurysms primarily in abdominal organs. It is an acute, non-inflammatory, non-arteriosclerotic degenerative disease, frequently associated with ruptured aneurysms and intra-abdominal hemorrhage requiring immediate surgery. An increasing number of cases with this condition have been reported in the literature [1]. Although there are numerous reports of SAM-related ruptured aneurysms of abdominal organ arteries and intestinal ischemia, there are only a limited number of investigations on other abnormalities of abdominal organs related to SAM [2]. In this report we describe the case of a patient who developed renal infarction and pancreatic enlargement suspected to be associated with SAM.

## Case presentation

A 52-year-old Japanese man with no significant prior medical history and unknown smoking habits was referred to a urologist for investigation of hematuria, and was diagnosed as having urinary bladder cancer after further examination. He underwent three sessions of transurethral resection of the bladder tumor and intra-vesical instillation therapy (doxorubicin and cacillus Calmette-Guerin) and was then placed under observation. During the follow-up period no abdominal symptoms were observed, and an abdominal computed tomography (CT) scan revealed the incidental finding of dissection of the superior mesenteric artery (SMA). Our patient was then referred to our hospital.

**Figure 1:**
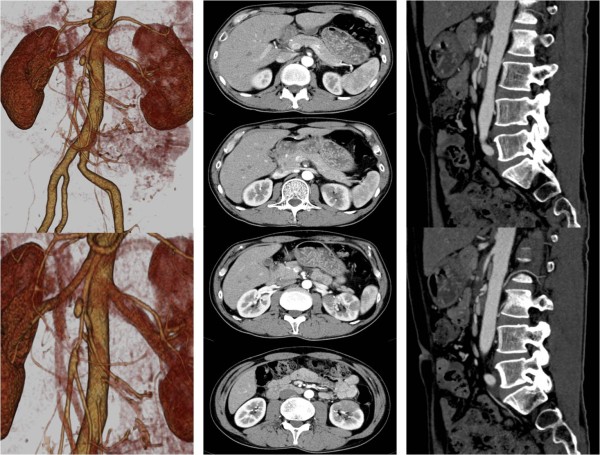
**Computed tomography at the time of first examination (left, three-dimensional construction; middle, transverse plane; right, sagittal plane).** A dissecting arterial aneurysm, with a maximum diameter of 12.3mm (compared with 6.6mm at an unaffected site), was localized approximately 1.5 and 5cm distal to the ostium of the superior mesenteric artery. A false lumen was partially occupied by a thrombus. Distal to the occlusion, the arterial wall was irregularly shaped, with narrow and wide sections. An irregular shaped arterial wall was also observed in the inferior mesenteric artery. Arterial dissection with thromboembolism was also observed in the left renal artery, accompanied by ipsilateral renal infarction.

His physical examination findings on admission were height of 176cm, weight of 64.5kg, blood pressure of 111/69mmHg, and pulse rate of 70bpm. Our patient had no fever and no other abnormal physical findings such as abdominal symptoms. His blood biochemistry test results were also normal. A contrast-enhanced abdominal CT scan revealed an arterial aneurysm and a dissection located approximately 1.5 and 5cm distal to the ostium of the SMA (Figure 1). A false lumen of the artery was partially occluded by a thrombus. Distal to this occlusion, the arterial wall was irregularly shaped with both narrow and wide sections. The inferior mesenteric artery was also observed to have an irregular arterial wall. The renal artery also showed dissection, with an occluded false lumen; an ipsilateral renal infarction was observed as well.

**Figure 2:**
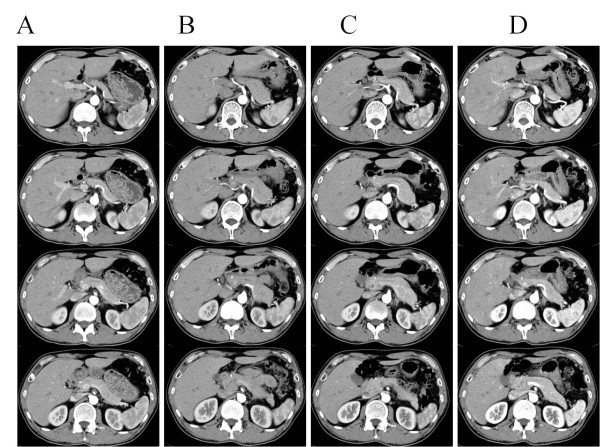
**Pancreatic enlargement ((A), first examination; (B-D), two months, three months and one year later, respectively).** At two months, computed tomography revealed an enlargement of the pancreatic tail adjacent to the splenic artery. However, this enlargement of the pancreas gradually improved.

**Figure 3:**
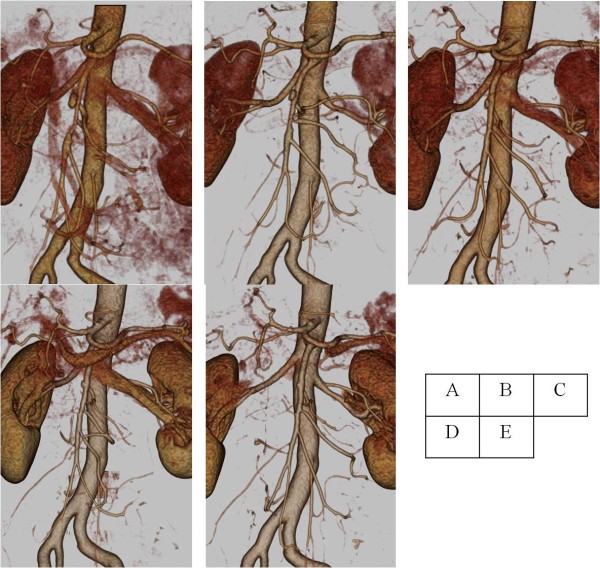
**Change in the superior mesenteric artery ((A), first examination; (B-E), one month, two months, three months and one year later, respectively).** Three-dimensional computed tomography showed a gradual re-expansion of the true lumen and improvements in the irregular arterial wall.

**Figure 4:**
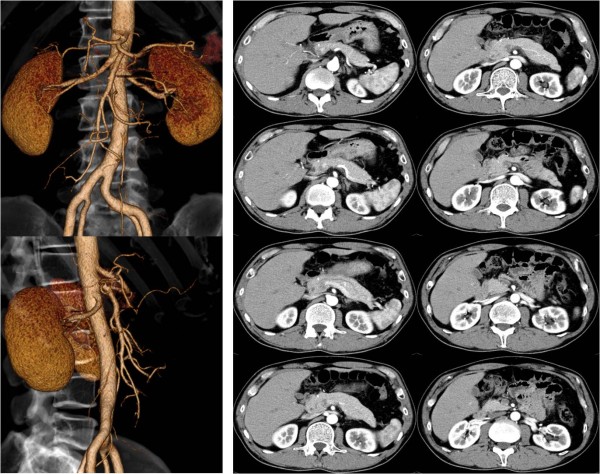
**Follow-up computed tomography.** The majority of changes continued to improve to almost normal over the next two-and-a-half years.

On the basis of good CT contrast enhancement of the artery distal to the dissected portion and the absence of increased inflammatory parameters or clinical symptoms, our patient was prescribed oral aspirin and placed under observation. After two months, a follow-up CT scan revealed an enlargement of the pancreatic tail adjacent to the splenic artery (Figure 2). In view of the absence of clinical symptoms or elevated levels of pancreatic enzymes, our patient was again placed under observation. Follow-up was carried out using three-dimensional CT scans, which showed re-expansion of the true lumen, gradual improvement of the arterial wall irregularities, and reduction of the renal infarction and pancreatic enlargement (Figure 3). These changes continued to improve to almost normal over the following 15 months (Figures 2, 3 and 4). No findings suggestive of a recurrence of the disorder have been observed in our patient in the three years after his initial presentation.

## Discussion

SAM is a non-inflammatory, non-arteriosclerotic degenerative disease that was first reported in 1976. It was initially named segmental mediolytic arteritis, but was subsequently renamed SAM in 1995 [1]. SAM commonly affects the arteries of abdominal organs, with a number of cases requiring emergency surgery for intra-abdominal hemorrhage resulting from ruptured aneurysms and intestinal infarction. Regardless, it is associated with good prognosis [3]. SAM may also be accompanied by coronary arterial lesions or intra-cranial vascular lesions [4].

The pathological characteristics of SAM include foamy changes in arterial smooth muscle cells, followed by mediolysis and gap formation with exudation and fibrin deposition. These changes are followed by rupture of the tunica interna and dissection of the arterial wall. The remaining tunica externa is distended to form an aneurysm. The walls of the aneurysm contain characteristic islet-like residues of the tunica media. SAM is classified as a non-inflammatory condition due to the absence of local infiltration of inflammatory cells into the lesion in some cases and the absence of vascular deposition of immunoglobulins or complement.

The first Japanese case of SAM was reported in 1992. Subsequently, several other case reports have been published. In Japanese patients, SAM commonly affects middle-aged adults, with men being twice as likely to be affected as women. The majority of the aneurysms are of the dissecting type, with about 35% of all cases involving multiple aneurysms [3,5]. The proposed pathogenesis of SAM involves vasoactive substances, such as catecholamines and endothelin, inducing vasospasm that causes degeneration of vascular medial smooth muscle [2]. Factors suggested to mediate this pathological condition include autoimmune diseases, hypoxia, and shock. However, the exact cause has yet to be elucidated. Similarly, the pathogenesis of multiple lesions remains unknown. None of these factors appear to have been involved in our patient’s case, and therefore the pathogenesis remains unknown.

Other diseases and conditions associated with the formation of aneurysms and/or dissections in abdominal organ arteries include arteriosclerosis, infection, aortitis syndrome, polyarteritis nodosa, cystic medial degeneration, fibromuscular dysplasia (FMD), pregnancy, and trauma. Of these, FMD has been the most extensively discussed. Lie *et al*. proposed that SAM was a subtype of FMD on the basis that both SAM and FMD produce the ‘string of beads’ appearance on angiography [6]. However, Slavin *et al*. argued that although some types of SAM produce lesions such as fibrosis of granulation tissue within arteries that can be regarded as a precursor lesion of FMD, not all types of FMD precede SAM [1]. FMD primarily produces stenotic lesions and commonly affects the renal and carotid arteries of younger women, including children. In addition, while a ruptured aneurysm in SAM results from mediolysis, a ruptured aneurysm in FMD is thought to result from atrophy or disappearance of the tunica media. The precise mechanism of this change is yet to be established. These findings support the argument of Slavin *et al.*[5]. In our patient’s case, we did not perform a pathological examination and therefore were unable to clarify this point.

While most reported cases of SAM involve ruptured aneurysms, lesions that do not rupture have hyperplasia of granulation tissue in the defect formed by mediolysis. At the same time, similar changes occur around the hematoma, which include a thrombus being formed and then organized, followed by absorption and shrinkage of the hematoma. While some reports describe cases in which an aneurysm resolved spontaneously after multiple angiography sessions [7], others describe cases of SAM in which irregularities in the shape of the arterial wall remained unresolved. Therefore, the natural history of SAM is currently unknown and as a consequence there are no reports on medical therapy for this condition.

Treatment selection in our patient’s case was based on previous case reports identified by a search using ‘SMA dissection’ as the keyword. This choice was based on the marked similarity of findings between SMA dissection and our patient’s case. These similarities included a lesion present between the root and proximal part of the SMA, formation of the aneurysm and uncommon rupture, and the presence of a false lumen that remained open, as occurs in about one-half of reported cases. The reported treatment options for SMA dissection include conservative treatment, anticoagulants, endovascular treatment, and surgical treatment, although there is no general agreement on these treatment options. In light of recent findings that suggested early introduction of anti-platelet therapy may reduce the need for surgery, we chose this course of treatment in our patient’s case. Although SMA dissection is associated closely with hypertension and smoking [8], it is considered a different condition from SAM as arterial narrowing is not found on imaging. Our patient also had no prior history of hypertension or smoking.

The literature contains an interesting case in which all the veins running adjacent to the artery affected by SAM exhibited varying degrees of changes, such as edema, dissection, lysis and defects in the muscle layer, and hyperplasia of elastic fibers [9]. The pathogenesis of these venous changes, including whether or not they occurred secondary to the arterial lesions, remains unknown. In our patient’s case the pancreatic enlargement was observed only at the part of the organ that was in contact with the splenic artery. This may be a similar situation to the venous changes described above. As SAM can occur in arteries of multiple systems at various points in time, it is possible that the pancreatic lesion was present, but not detected at the initial presentation. The absence of clinical symptoms, diffuse lesions, or pancreatic duct stenosis suggests that the pancreatic change observed was different from autoimmune-mediated pancreatitis.

In our patient’s case, the middle colic artery, which is most closely associated with rupture and ischemia, was derived from the true lumen on the dorsal side of the dissected part. This may have contributed to the absence of clinical symptoms and the development of pancreatic enlargement as a secondary change around the affected artery. Three-dimensional CT was useful for follow-up evaluation [10] and showed our patient had a very rare case of SAM accompanied by various changes.

## Conclusions

The lesions observed in our patient’s case were considered to be associated with SAM as they included irregularities in arterial walls, and the development of an aneurysm and dissections in multiple arterial systems that resolved spontaneously. Our patient’s case constitutes a very rare example of SAM accompanied by organ involvement, including renal infarction and pancreatic enlargement. Three-dimensional CT was useful for follow-up evaluation in our patient.

## Consent

Written informed consent was obtained from the patient for publication of this case report and any accompanying images. A copy of the written consent is available for review by the Editor-in-Chief of this journal.

## Competing interests

The authors declare that they have no competing interests.

## Authors’ contributions

NI was a major contributor in writing the manuscript. GK, YS and HT contributed to the conception and interpretation of the disease on the basis of several articles. All authors read and approved the final manuscript.
